# Clinicopathological Characteristics and Prognosis of Papillary Thyroid Carcinoma in Naturally Menopausal Women with Various Durations of Premenarche, Reproductive Periods, and Postmenopausal Stages

**DOI:** 10.1155/2017/5702716

**Published:** 2017-03-03

**Authors:** Xuhang Zhu, Bin Yu, Yu-qing Huang, Jing-nan Zhou, Ming-Hua Ge

**Affiliations:** ^1^Second Clinical Medical College, Zhejiang Chinese Medical University, Binjiang District, Hangzhou 310022, China; ^2^Department of Head and Neck Surgery, Zhejiang Province Cancer Hospital, Gongshu District, Hangzhou 310022, China; ^3^Department of Equipment, Zhejiang Province Cancer Hospital, Gongshu District, Hangzhou 310022, China; ^4^Department of Head and Neck Surgery, Zhejiang Province Cancer Hospital, Gongshu District, Hangzhou 310022, China

## Abstract

*Background.* Papillary thyroid cancer (PTC) exhibits a higher incidence in women. Due to various ages at menarche and menopause, estrogen levels vary, which may account for the differences in the occurrence, development, and prognosis of female patients with PTC. *Objective.* The aim of this study was to investigate the association between various durations in different estrogen levels and PTC and to provide important information to guide clinical management and treatment of this disease. *Methods.* First, we selected naturally menopausal female study subjects diagnosed with PTC at Zhejiang Cancer Hospital from 2007 to 2012 and then compared the differences in clinicopathological characteristics and prognosis among subjects with various lengths of premenarche, reproductive periods, and postmenopausal stages. *Results.* We found that all patients showed a significantly higher incidence of tumor multicentricity and intrathyroidal dissemination as the time after menopause increased. Additionally, women with shorter (<30) or longer (>38) reproductive lives had increased recurrence rates of PTC. *Conclusions.* In this study, we did not find any relationship of self-reported menarche and menopausal ages with the prognosis of PTC patients. More importantly, natural postmenopausal PTC patients with shorter or longer reproductive life, compared to the normal groups, had a higher rate of cancer recurrence and the patients with these characteristics could be recommended a more aggressive surgical treatment.

## 1. Introduction

Thyroid cancer is a more predominant malignancy in women than in men [1], which is likely due to the more variable hormonal environments in women than in men [2]. Additionally, the incidence of thyroid cancer is equal in prepubertal girls and boys and increases in females by up to 14 times after the onset of menstruation [3]. Jonklaas et al. [4] found that postmenopausal women diagnosed with papillary thyroid cancer (PTC) had worse outcomes than premenopausal women and suggested that menopause-associated hormonal alterations may be the cause of this outcome, which was supported by other studies [5, 6]. Some studies have focused on the role of estrogen receptors and estradiol in PTC, which have suggested that estradiol can stimulate the proliferation of PTC cells [7–9]. These data showed that the occurrence, development, and prognosis of PTC are significantly influenced by the levels of sex hormones during a woman's life.

Understanding the relationship between menstrual characteristics and PTC could be beneficial for better surgical management and treatment of PTC patients. Menarche and menopause are two peculiar aspects of a woman's menstrual characteristics. Therefore, a woman's lifespan can be divided into three stages (the premenarche stage, reproductive period, and postmenopausal stage), and each stage can be divided into three parts, depending on standard references. The most important indicator is the length of each stage, measured in years, which is a measure of exposure to estrogen. Most studies have addressed the relationship between PTC and the common parameters of menstrual characteristics such as age at menarche or menopause [10–12], but few studies have evaluated varying durations of estrogen exposure, especially among female Chinese patients.

In this retrospective study, we enrolled patients who had undergone natural menopause among 3790 female patients diagnosed with PTC at Zhejiang Cancer Hospital between 2007 and 2012. We investigated differences in the clinicopathological characteristics and prognosis among female patients with various estrogen exposures to provide important information to guide clinical management and treatment.

## 2. Methods

### 2.1. Ethics Statement

All participants provided written informed consent, and the study protocol was approved by the Ethics Committee at Zhejiang Cancer Hospital.

### 2.2. Study Population

Between January 2007 and December 2012, 3790 female patients underwent initial treatment for PTC in the Department of Head and Neck Surgery of Zhejiang Cancer Hospital. In total, 429 patients who experienced natural menopause, underwent primary surgical treatment in our hospital, and were diagnosed with PTC were enrolled in this study. The operation was performed by a surgical team, and at least two pathologists reviewed the pathological findings. Individuals who had previous and recent histories of neck surgeries, artificial menopause, hysterectomy, ovariectomy, and drug intake for a long period of time were excluded, as these factors can influence hormone levels. Patients with diseases affecting natural menstruation (e.g., oophoroma or chronic diseases such as tuberculosis and malignancy) were also excluded from this study.

### 2.3. Menstrual Variables

The length of premenarche was established by the age at menarche. The age at the time of each patient's first period was considered the age at menarche; the time from menarche to menopause was defined as a woman's natural reproductive span (also called length of reproductive life), and the number of years since menopause was calculated from the age at menopause to the age at diagnosis. The judgment of menopausal status depended on the definition of the World Health Organization, which specifies cessation of menstruation for at least 12 months. All information about menstruation was reconfirmed by telephone.

Each stage was divided into three portions; the length of premenarche and the reproductive span was divided based on the Shanghai Women's Health Study, which is a large, population-based cohort study conducted in China. No standard was provided for the number of years since menopause, and the criteria for the three grades of the other categories were as follows: below the 25th percentile, between the 25th and 75th percentiles, and above the 75th percentile of the total population.

Based on this information, the menstrual variable categories used as the reference groups in our analyses were as follows: aged 14–16 years at menarche, 30–38 reproductive years, and 4–14 years after menopause.

### 2.4. Outcome Definition

The evaluation of clinicopathological features was performed by professional pathologists. The tumor/node/metastasis (TNM) classification was estimated according to the 2010 AJCC criteria. Follow-up was performed after treatment was completed in our hospital and before December 31, 2015. Clinical examinations, blood parameter tests, and ultrasonography (USG) were performed in all patients every 3 months during the first year and every 6 months during the second year. A chest X-ray or CT scan was performed once each year. Recurrence was confirmed by FNAB and reoperation if any suspicion of a malignancy was found by imaging. Information on the disease-specific survival of patients or patients who did not undergo subsequent treatment in our hospital was confirmed by phone contact or letters.

### 2.5. Measurement of Selected Potential Confounders

Information regarding baseline conditions was collected from the medical record data in our hospital and included age (years), TNM stages (I, II, III, and IV), tumor size (≤1 cm, >1 cm), multicentricity (solitary, multiple), bilaterality (unilateral, bilateral), intrathyroidal dissemination (present, absent), thyroid nodular goiter (present, absent), Hashimoto's thyroiditis (present, absent), operation on primary tumor (total thyroidectomy, subtotal thyroidectomy), and lymph node dissection (not done, central node dissection, and total node dissection), iodine radiotherapy (done, not done), and time of pregnancy (age at first birth, age at last birth).

### 2.6. Statistical Analysis

The chi-square test and Fisher's exact test were used to compare clinicopathological characteristics among the subgroups, and the Kaplan-Meier method and log-rank test were used to analyze the time-dependent variables. Prognostic factors that were significant in the univariate analysis were further evaluated using the multivariate Cox model test for independent significance. These analyses were performed using SPSS version 12.0 (SPSS Inc., Chicago, IL, USA). *P* values < 0.05 were considered significant.

## 3. Results

Table 1 shows the characteristics of the study population. At the time of diagnosis, the age of our study subjects ranged from 44 to 80 years, with a median of 57 years. The median premenarche period, length of reproductive span, and number of years since menopause were 15, 35, and 8 years, respectively. The age at first birth and last birth of our study subjects ranged from 16 to 35 years and from 18 to 44 years, with a median of 24 and 30 years. The follow-up periods ranged from 36 to 107 months (median, 54 months). The incidences of cancer recurrence and diseases were 4.0% and 0.6%, respectively. Additionally, the recurrence rates of metastasis to the cervical lymph nodes, metastasis to the residual thyroid tissues, and distant metastasis were 64.7%, 23.5%, and 11.7%, respectively.

Next, we analyzed the pathological data for PTC and papillary thyroid microcarcinoma (PTMC) (Tables 2 and 3). We found no significant differences among most clinicopathological features in each stage of hormone exposure, and a significant difference in age was observed among the patients in the three stages of the reproductive span and the postmenopause period (*P*^trend^ < 0.05). Significant differences related to multicentricity, intrathyroidal dissemination, and recurrence of disease were found in the three stages of postmenopause. As the postmenopausal period increased, age at first birth, the proportion of patients with multiple nodules, intrathyroidal dissemination, and the recurrence of diseases also increased (*P*^trend^ < 0.05). As the length of reproductive life increased, age at last birth increased (*P*^trend^ < 0.05). Few patients died of the disease in our study. Based on this result, we further analyzed the clinicopathological features of PTMC patients in three menstrual stages. The differences in age at first birth, age at last birth, intrathyroidal dissemination, and recurrence among subjects in the three postmenopausal stages were not significant, and the other outcomes were similar to those of the PTC patients.

Table 4 represents univariate and multivariable-adjusted HRs of the recurrence according to the multicentricity, intrathyroidal dissemination, age at menarche, length of reproductive life, years after menopause, age at first birth, and age at last birth. A shorter (<30 years) or longer (>38 years) reproductive span was associated with recurrence in PTC patients. Compared to the reference group, the HRs and 95% CIs were 3.4 (1.0, 11.3) for women with a span of <30 years and 4.6 (1.5, 13.9) for women with a span of >38 years in the univariate model. The HRs and 95% CIs were 4.2 (1.2, 13.9) for women with a span of <30 years and 5.6 (1.7, 17.2) for women with a span of >38 years in the multivariable model. In PTMC patients, compared to the reference group, the HRs and 95% CIs were 4.5 (1.2, 16.9) for women with an older age at menarche and 4.4 (1.1, 18.7) for a span of <30 years in the univariate model, and the HRs and 95% CIs were 4.5 (1.2, 16.9) for women with an older age at menarche and 4.3 (0.8, 23.1) for a span of <30 years in the multivariable model. The age at menarche and length of reproductive span were not significant independent factors (*P* > 0.05) in PTMC patients. The reproductive span (*P* = 0.00) was the independent factor that influenced the recurrence of the disease, and no other factors were found to be significant in PTC patients in the current study. The interaction between menstrual stages and other factors in PTC patients was not significant (data not shown).

**Figure 1:**
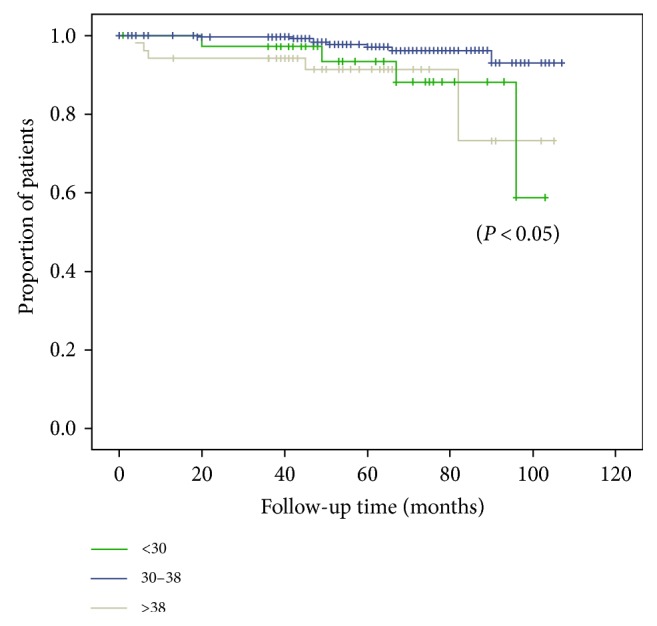
Recurrence of PTC according to length of reproductive span.

The Kaplan-Meier survival analysis revealed that compared to the reference values, a significant difference in recurrence existed (10.3% versus 2.4% versus 9.4%; *P* < 0.05; Figure 1), and women with a shorter (<30 years) or longer (>38 years) reproductive span had a higher risk of PTC recurrence.

## 4. Discussion

In this study, all patients showed a significantly higher incidence of tumor multicentricity and intrathyroidal dissemination with increasing time after menopause. Additionally, our results demonstrated that the reproductive span length was an independent factor that influenced the prognosis of PTC. Women with shorter or longer reproductive spans had a higher risk of recurrence than the reference group.

The incidence of tumor multifocality and intrathyroidal dissemination, which represented invasive behavior by a tumor and led to a worse prognosis and the need for more aggressive treatments than unilateral tumors [13], was increased as the number of years after menopause increased in our study. However, we found that they were not the factors that influenced the prognosis of PTC.

It was controversial whether there were positive associations between thyroid cancer and time of pregnancy. Memon et al. [14] found increasing tendency of risk with increasing age at last pregnancy; on the other hand, Kabat et al. [12] found that women who had a first live birth with age between 20 and 24 years also had a significant risk of papillary thyroid cancer. Actually, according to many published reports [12, 14–16] along with our present study, we did not get any significant positive associations between time of pregnancy and PTC.

Although many studies [17–19] have suggested that older age, tumor size, and advanced stage are risk factors of cancer recurrence, we found only a positive association of reproductive span with the prognosis of PTC in our study, and we did not find other factors that influence the prognosis of PTC.

Although epidemiological and experimental studies have suggested a potential association between the development of thyroid malignancies and estrogen, this conclusion is not understood [20]. Rajoria et al. [21] documented that estrogen was closely related to increased adherence, invasion, and migration of thyroid cancer cell lines. In our study, we found that the patients with a longer reproductive life have higher risk of cancer recurrence compared to the normal. Longer reproductive life may reflect the status of women with relatively higher levels of estrogen, which influences the development and progression of PTC cells, and it has already been reported that estradiol stimulates the proliferation of PTC cells in vitro [7–9, 21–23]. In malignant and benign thyroid cells, estradiol (E2) by its membrane-bound receptor (mER) also stimulated activation of the MAP kinase signaling pathway [21–25]. Additionally, via mER, E2 activated the phosphatidylinositol 3-kinase (PI3K) pathway [26]. Both the MAPK and the PI3K pathways are significant for the proliferation and propagation of thyroid cancer. On the other hand, better prognoses have been observed in women before menopause with higher estrogen levels than postmenopausal patients [27]. Additionally, studies in Mexican patients [28, 29] have reported that the prognosis worsens in women over 50 years old. These reports support an opinion first expressed by Jonklaas and colleagues [4], in which the prognosis of patients in an estrogen-deficient environment is worse than that of patients diagnosed when they were exposed to female hormones. In our study, the patients with a shorter reproductive life have higher risk of cancer recurrence compared to the normal reproductive life, because shorter reproductive life may reflect the status of women with relatively low levels of estrogen. Schiff and Walsh [30] found that, in an estrogen-deficient environment, estradiol decreases and follicle-stimulating hormone (FSH) plasma level increases (>50 mIU/mL). An increased level of FSH as well as the absence of estradiol induces the higher epidermal growth factor (EGFR) mRNA expression; elevated EGFR activity initiates the DNA synthesis and cell proliferation by converging with the estrogen receptors, resulting in the development of cancer [31, 32]. Our study revealed that the relationship between the length of reproductive span and recurrence was “U shaped,” and it showed that only in the abnormal reproductive span did the PTC patients have a higher risk of cancer recurrence. Additionally, no significant association was observed between age at menarche or the number of years after menopause and the prognosis of PTC patients. It seemed that the prognosis of PTMC was unrelated to the menstrual stage, which may have been due to the selection bias of the study population or the lack of samples. These results might play an important role in guiding the primary surgery, application of adjuvant therapy, and follow-up protocol for naturally postmenopausal patients.

Although PTC patients have a good prognosis, the rate of recurrence was 8–23% as reported by Kim et al. [33]; however, the recurrence rate in our study was 4.0%, which was different from previous studies. Additionally, 80–90% of the recurrences were local; 75% of the recurrence cases were reported to metastasize to cervical lymph nodes, and the remaining recurrence cases occurred in the remaining thyroid tissue [34, 35]. These findings were similar to those in our study. However, no significant difference was found among the treatments in various stages of menstruation, reproductive spans, and postmenopause in our study. PTC is known to have a good prognosis, but for women with PTC with natural menopause, we recommended a more personalized or aggressive treatment according to the menstrual span.

The strengths of this study included the following. The current study was restricted to women who had undergone natural menopause without drugs or diseases that affect female hormones. In contrast to previous studies, we considered the entire menstrual history of women instead of the ages at menarche and menopause because these ages cannot accurately evaluate the effect of female hormones on PTC. We used the length of the menstrual span as a measure of exposure to estrogen. To the best of our knowledge, the histological examination of estrogen receptors in PTC patients is not widespread, but the history of patients' menstrual characteristics can be easily and conveniently obtained. Moreover, the association between the reproductive span and PTC recurrence may be a novel finding, which may be beneficial to surgical treatment. More studies are needed to confirm this finding.

The main limitation of this study was that the information about menstrual history was self-reported and may be influenced by recall bias. However, previous studies have shown that the recall of ages at menarche and menopause is relatively reliable [36–38]. Additionally, differences were observed between individuals due to environment and lifestyle, and the findings of our study may not be generalizable to other populations. Another limitation was the small number of patients studied; due to the excellent prognosis of PTC patients, the numbers of cases of recurrence or death are particularly small. Therefore, extensive research on a larger population is required to confirm our conclusions and to develop a more precise standard for naturally postmenopausal patients.

## 5. Conclusion

In this study, we did not find any relationship of self-reported menarche and menopausal ages with the prognosis of PTC patients. More importantly, natural postmenopausal PTC patients with shorter or longer reproductive life, compared to the normal groups, had a higher rate of cancer recurrence, and the patients with these characteristics could be recommended a more aggressive surgical treatment.

## Figures and Tables

**Table 1:** Characteristics of the study population.

Variables	Median (range)
Age at diagnosis (years)	57 (44–80)
Age at menarche (years)	15 (10–19)
Length of reproductive life (years)	35 (16–45)
Years postmenopause (years)	8 (0–36)
Time of pregnancy (years)	
Age at first birth	24 (16–35)
Age at last birth	30 (18–44)
Follow-up time (months)	54 (36–107)

Recurrence of cancer	Case, *n* = 429 (%)
Present	17 (4.0)
Absent	412 (96.0)

Location of recurrence	Case, *n* = 17 (%)
Residual thyroid tissues	11 (64.7)
Cervical lymph nodes	4 (2.5)
Distant metastasis	2 (11.7)

Dead of cancer	Case, *n* = 429 (%)
Present	3 (0.6)
Absent	426 (99.4)

**Table 2:** Clinicopathological characteristics, treatment modalities, and outcome characteristics of PTC patients at various menstrual stages.

Variables	Total (*n* = 429)				Total (*n* = 429)				Total (*n* = 429)			
	Age at menarche, years			*P* ^a^ for trend	Length of reproductive life, years			*P* ^a^ for trend	Years after menopause, years			*P* ^a^ for trend
	Group 1 (*n* = 37): <14	Group 2 (*n* = 291): 14–16	Group 3 (*n* = 101): >16		Group 1 (*n* = 39): <30	Group 2 (*n* = 337): 30–38	Group 3 (*n* = 53): >38		Group 1 (*n* = 94): <4	Group 2 (*n* = 225): 4–14	Group 3 (*n* = 110): >14	
*Age (mean, range)*	55.8 (37–78)	58.6 (42–80)	56.9 (47–74)	NS	54.2 (37–74)	57.9 (46–78)	60.7 (54–80)	**0.00**	51.8 (46–70)	56.3 (46–78)	66.7 (46–77)	**0.00**
Age at first birth (mean, range)	24.1 (16–33)	24.3 (16–35)	25.1 (18–35)	NS	23.0 (16–34)	24.6 (16–35)	24.8 (16–34)	NS	23.4 (16–35)	24.8 (16–35)	24.7 (16–35)	**0.04**
Age at last birth (mean, range)	29.2 (18–36)	29.8 (17–44)	30.7 (21–43)	NS	27.9 (17–39)	30.2 (17–44)	29.8 (18–42)	**0.03**	29.1 (17–41)	30.1 (18–43)	30.5 (17–44)	NS

*Maximal tumor diameter*												
≤1 (cm) >1 (cm)	27 10	194 97	70 31	NS	26 13	229 108	36 17	NS	68 26	156 69	67 43	NS

*Multicentricity*												
Solitary Multiple	23 14	199 92	65 36	NS	23 16	230 107	34 19	NS	69 25	154 71	64 46	**0.02**

*Bilaterality*												
Unilateral Bilateral	29 8	225 66	75 26	NS	30 9	262 75	37 16	NS	77 17	172 53	80 30	NS

*Extrathyroidal extension*												
Present Absent	9 28	127 164	42 59	NS	13 26	149 188	16 37	NS	34 60	92 133	52 58	NS

*Intrathyroidal dissemination*												
Present Absent	2 35	15 276	6 95	NS	5 34	15 322	3 50	NS	2 92	10 215	11 99	**0.01**

*Thyroid nodular goiter*												
Present Absent	22 15	173 118	58 43	NS	17 22	205 132	31 22	NS	54 40	136 89	63 47	NS

*Hashimoto's thyroiditis*												
Present Absent	3 34	9 282	6 95	NS	2 37	15 322	1 52	NS	7 87	10 215	1 109	NS

*TNM stage*												
I	23	173	59	NS	27	198	30	NS	58	135	62	NS
II	1	10	2	NS	1	9	2	NS	2	6	4	NS
III	8	57	18	NS	8	66	10	NS	17	45	22	NS
IV	5	51	22	NS	3	64	11	NS	17	39	22	NS

*T staging*												
T1	29	225	83	NS	32	263	42	NS	79	180	78	NS
T2	2	22	3	NS	1	22	4	NS	6	12	9	NS
T3	4	32	8	NS	6	35	3	NS	9	20	15	NS
T4	2	12	7	NS	0	17	4	NS	0	13	8	NS

*N staging*												
N0/Nx	25	197	62	NS	28	221	35	NS	63	149	72	NS
N1a	9	55	21	NS	7	66	12	NS	16	50	19	NS
N1b	3	39	18	NS	4	50	6	NS	15	26	19	NS

*M staging*												
M0	37	287	101	NS	39	334	52	NS	93	224	108	NS
M1	0	4	0		0	3	1		1	1	2	

*Operation of primary tumor*												
Total thyroidectomy	11	101	43	NS	18	117	20	NS	32	80	43	NS
Subtotal thyroidectomy	26	190	58		21	220	33		62	167	67	

*Lymph node dissection*												
Not done	3	39	9	NS	6	39	6	NS	12	34	15	NS
Central node dissection	27	200	73	NS	29	232	39	NS	63	188	70	NS
Total node dissection	7	52	19	NS	4	66	8	NS	19	38	25	NS

*Iodine radiotherapy*												
Done Not done	6 31	31 260	12 89	NS	5 34	37 300	7 46	NS	12 82	24 201	13 97	NS

*Recurrence of disease*												
Present Absent	2 35	11 280	4 97	NS	4 35	8 329	5 48	NS	0 94	10 215	7 103	**0.02**

*Disease-specific survival (DSS)*												
Dead Alive	1 36	2 289	0 101	NS	1 38	1 336	1 52	NS	0 94	1 224	2 107	NS

^a^ANOVA for continuous variables and chi-square test for categorical variables. NS: not significant. Bold values are less than or close to 0.05.

**Table 3:** Clinicopathological characteristics, treatment modalities, and outcome characteristics of PTMC patients at various menstrual stages.

Variables	Total (*n* = 291)				Total (*n* = 291)				Total (*n* = 291)			
	Age at menarche, years			*P* ^a^ for trend	Length of menstrual life, years			*P* ^a^ for trend	Years after menopause, years			*P* ^a^ for trend
	Group 1 (*n* = 27): <14	Group 2 (*n* = 194): 14–16	Group 3 (*n* = 70): >16		Group 1 (*n* = 26): <30	Group 2 (*n* = 229): 30–38	Group 3 (*n* = 36): >38		Group 1 (*n* = 68): <4	Group 2 (*n* = 156): 4–14	Group 3 (*n* = 67): >14	
*Age (mean, range)*	54.9 (37–68)	57.9 (44–77)	56.8 (45–74)	NS	55.3 (37–72)	57.3 (46–77)	59.6 (55–70)	**0.02**	52.1 (44–63)	56.3 (37–68)	65.3 (55–77)	**0.00**
Age at first birth (mean, range)	24.5 (16–33)	24.1 (16–35)	24.6 (18–35)	NS	22.8 (16–32)	24.4 (16–35)	24.8 (16–34)	NS	23.3 (16–35)	24.6 (16–35)	24.7 (16–35)	NS
Age at last birth (mean, range)	29.9 (18–36)	29.4 (17–42)	30.1 (21–43)	NS	27.2 (17–37)	29.8 (17–43)	30.2 (18–42)	NS	29.0 (17–40)	29.6 (18–43)	30.3 (17–41)	NS

*Multicentricity*												
Solitary Multiple	17 10	140 54	47 23	NS	14 12	165 64	25 11	NS	52 16	110 46	42 25	**0.02**

*Bilaterality*												
Unilateral Bilateral	22 5	160 34	55 15	NS	21 5	190 39	26 10	NS	58 10	126 30	53 14	NS

*Extrathyroidal extension*												
Present Absent	7 20	52 142	21 49	NS	5 21	69 160	6 30	NS	18 50	45 111	17 50	NS

*Intrathyroidal dissemination*												
Present Absent	1 26	4 190	2 68	NS	1 25	6 223	0 36	NS	0 68	5 151	2 65	NS

*Thyroid nodular goiter*												
Present Absent	19 8	125 69	44 26	NS	13 13	153 76	22 14	NS	46 22	99 57	43 24	NS

*Hashimoto's thyroiditis*												
Present Absent	1 26	8 186	4 66	NS	1 25	11 218	1 35	NS	5 63	7 149	1 66	NS

*TNM stage*												
I	20	154	49	NS	22	174	27	NS	53	114	56	NS
II	0	0	0	NS	0	0	0	NS	0	0	0	NS
III	7	28	13	NS	3	39	6	NS	10	32	6	NS
IV	0	12	8	NS	1	16	3	NS	5	10	5	NS

*T staging*												
T1	26	187	70	NS	26	221	36	NS	68	149	66	NS
T2	0	0	0	NS	0	0	0	NS	0	0	0	NS
T3	1	6	0	NS	0	7	0	NS	0	7	0	NS
T4	0	1	0	NS	0	1	0	NS	0	0	1	NS

*N staging*												
N0/Nx	20	157	50	NS	22	177	28	NS	54	117	56	NS
N1a	7	27	13	NS	3	37	7	NS	10	31	6	NS
N1b	0	10	7	NS	1	15	1	NS	4	8	5	NS

*M staging*												
M0	27	193	70	NS	26	229	35	NS	68	155	67	NS
M1	0	1	0		0	0	1		0	1	0	

*Operation of primary tumor*												
Total thyroidectomy	6	54	25	NS	10	64	11	NS	23	42	47	NS
Subtotal thyroidectomy	21	140	45		16	165	25		45	114	20	

*Lymph node dissection*												
Not done	2	31	9	NS	4	34	4	NS	12	20	10	NS
Central node dissection	22	148	53	NS	21	173	29	NS	51	122	50	NS
Total node dissection	3	15	8	NS	1	22	3	NS	5	14	7	NS

*Iodine radiotherapy*												
Done Not done	2 25	7 187	2 68	NS	1 25	8 221	2 34	NS	2 66	6 150	3 64	NS

*Recurrence of disease*												
Present Absent	1 26	5 189	4 66	NS	3 23	5 224	2 34	NS	0 68	8 148	2 65	NS

*Disease-specific survival (DSS)*												
Dead Alive	0 27	1 193	0 70	NS	1 25	0 229	0 36	NS	0 68	0 156	1 66	NS

^a^ANOVA for continuous variables and chi-square test for categorical variables. NS: not significant. Bold values are less than or close to 0.05.

**Table 4:** Univariate and multivariable-adjusted HRs (95% CI) of recurrence according to multicentricity, intrathyroidal dissemination, various menstrual stages, and time of pregnancy.

	PTC									PTMC								
Variables	Recurrence of disease: present/absent	Univariate HR	95% CI	*P*	*P* ^trend^	^∗^Multivariate HR	95% CI	*P*	*P* ^trend^	Recurrence of disease: present/absent	Univariate HR	95% CI	*P*	*P* ^trend^	^∗^Multivariate HR (††)	95% CI	*P*	*P* ^trend^
Multicentricity																		
Solitary Multiple	11/318 6/94	1.8	0.7–4.9	NS	NS	^a^1.6	0.5–5.5	NS	NS	9/228 1/53	0.5	0.1–4.2	NS	NS	^a^0.5	0.1–5.0	NS	NS

Intrathyroidal dissemination																		
Absent Present	9/242 8/170	1.2	0.5–3.2	NS	NS	^b^1.1	0.4–3.1	NS	NS	6/205 4/76	1.7	0.5–6.2	NS	NS	^b^2.8	0.7–12.6	NS	NS

Age at menarche (years)																		
<14	2/35	2.0	0.5–9.7	NS		^c^1.3	0.2–7.6	NS		1/26	1.7	0.2–14.3	NS		^c^1.7	0.2–14.3	NS	
14–16	11/280	1	Reference	NS	NS	^c^1	Reference	NS	NS	5/189	1	Reference	NS	NS	^c^1	Reference	NS	NS
>16	4/97	2.3	0.7–7.1	NS		^c^2.8	0.7–11.6	NS		4/66	4.5	1.2–16.9	0.02		^c^4.5	1.2–16.9	0.02	

Length of reproductive life (years)																		
<30	4/35	3.4	1.0–11.3	0.02		^d^4.2	1.2–13.9	0.00		3/23	4.4	1.1–18.7	NS		^d^4.3	0.8–23.1	NS	
30–38	8/329	1	Reference	0.04	NS	^d^1	Reference	0.02	NS	5/224	1	Reference	0.03	NS	^d^1	Reference	NS	NS
>38	5/48	4.6	1.5–13.9	0.01		^d^5.6	1.7–17.2	0.00		2/34	2.7	0.5–14.0	NS		^d^2.2	0.4–13.8	NS	

Years after menopause																		
<4	0/77	0.2	0.0–1.7	NS		^e^0.3	0.0–2.9	NS		0/52	0.0	0.0–1.8	NS		^e^0.0	0.0–4.2	NS	
4–14	11/243	1	Reference	NS	NS	^e^1	Reference	NS	NS	8/176	1	Reference	NS	NS	^e^1	Reference	NS	NS
>14	6/92	0.9	0.3–2.5	NS		^e^0.4	0.07–2.5	NS		2/53	1.0	0.2–3.7	NS		^e^0.8	0.1–4.6	NS	

Age at first birth																		
<21	3/75	1.0	0.2–4.3	NS		^f^1.0	0.2–4.2	NS		2/57	1.0	0.7–1.5	NS		^f^1.1	0.7–1.6	NS	
21–28	9/241	1	Reference	NS	NS	^f^1	Reference	NS	NS	6/161	1	Reference	NS	NS	^f^1	Reference	NS	NS
>28	5/96	1.5	0.3–6.4	NS		^f^1.4	0.3–6.8	NS		2/63	1.2	0.8–1.7	NS		^f^1.2	0.7–1.9	NS	

Age at last birth																		
<26	4/91	1.3	0.3–5.1	NS		^g^1.4	0.3–5.6	NS		3/70	0.9	0.6–1.3	NS		^g^0.9	0.6–1.4	NS	
26–34	9/238	1	Reference	NS	NS	^g^1	Reference	NS	NS	5/161	1	Reference	NS	NS	^g^1	Reference	NS	NS
>34	4/83	1.0	0.4–4.4	NS		^g^1.2	0.2–6.1	NS		2/50	1.0	0.7–1.5	NS		^g^0.9	0.6–1.5	NS	

PTC: papillary thyroid carcinoma; PTMC: papillary thyroid microcarcinoma; HR: hazard ratio; CIs: confidence intervals; *P*: *P* value for each variable; *P*^trend^: *P* value for the trend. ^∗^Adjusted for age, TNM stages, tumor size (except for ††), bilaterality, thyroid nodular goiter, Hashimoto's thyroiditis, surgery for primary tumor, lymph node dissection, multicentricity (except for a), intrathyroidal dissemination (except for b), age at menarche (except for c), length of reproductive span (except for d), years after menopause (except for e), age at first birth (except for f), and age at last birth (except for g).
