# Corrigendum to “Clinicopathological Characteristics and Prognosis of Papillary Thyroid Carcinoma in Naturally Menopausal Women with Various Durations of Premenarche, Reproductive Periods, and Postmenopausal Stages”

**DOI:** 10.1155/2018/8508059

**Published:** 2018-05-02

**Authors:** Xu-Hang Zhu, Bin Yu, Yu-qing Huang, Jing-nan Zhou, Ming-Hua Ge

**Affiliations:** ^1^Second Clinical Medical College, Zhejiang Chinese Medical University, Binjiang District, Hangzhou 310022, China; ^2^Department of Head and Neck Surgery, Zhejiang Province Cancer Hospital, Gongshu District, Hangzhou 310022, China; ^3^Department of Equipment, Zhejiang Province Cancer Hospital, Gongshu District, Hangzhou 310022, China

In the article titled “Clinicopathological Characteristics and Prognosis of Papillary Thyroid Carcinoma in Naturally Menopausal Women with Various Durations of Premenarche, Reproductive Periods, and Postmenopausal Stages” [1], the name of the first author was given incorrectly as Xuhang Zhu. The author's name should have been written as Xu-Hang Zhu. The revised authors' list is shown above.
